# Cutaneous clues to a rare osseous manifestation of paracoccidioidomycosis: The importance of early diagnosis

**DOI:** 10.1590/0037-8682-0232-2025

**Published:** 2025-10-17

**Authors:** Márcio Fellipe Menezes Viana, Rafael Fantelli Stelini, Andréa Fernandes Eloy da Costa França, Daniela Tsukumo Mitti, Luiz Fernando Monte Borella, Fabiano Reis

**Affiliations:** 1 Universidade estadual de Campinas, Campinas, SP, Brasil.

**Keywords:** Medical dermatology, Osteocutaneous involvement, Paracoccidioidomycosis

## Abstract

Paracoccidioidomycosis (PCM), the most prevalent systemic mycosis in Brazil, is caused by dimorphic fungi of the genus *Paracoccidioides* and is acquired primarily through inhalation. PCM is associated with a high mortality, particularly among men living in rural areas. This case report describes a 47-year-old man with osteocutaneous involvement diagnosed by biopsy and direct fungal examination. The osseous form of PCM is rare and should be differentiated from other infections and neoplasms. Management involves prolonged antifungal therapy and follow-up for at least two years. This case highlights the diagnostic value of dermatological assessments in atypical presentations of PCM.

## INTRODUCTION

Paracoccidioidomycosis (PCM) is the most prevalent systemic mycosis in Brazil and is caused by dimorphic fungi of the genus *Paracoccidioides*, primarily *P. brasiliensis* and *P. lutzii*. The fungus exists in a filamentous form in moist soil, with transmission occurring via inhalation of airborne propagules. This disease predominantly affects men living in rural areas. The incubation period is highly variable, ranging from days to several decades, owing to the organism’s ability to establish latent infection^1^ ^-^ ^5^.

PCM can affect any organ, and its clinical spectrum is typically classified into acute, subacute, and chronic forms. The acute/subacute (juvenile) form, more common in children and young adults primarily involves the mononuclear phagocytic system. Chronic PCM, which accounts for approximately 80% of cases, presents with slowly, progressive pulmonary involvement and mucocutaneous lesions, particularly affecting the upper aerodigestive tract and skin^1^ ^-^ ^5^.

Osseous involvement in PCM is uncommon. It typically appears as a round, well-demarcated, osteolytic lesion, with or without a sclerotic rim. Lesions may be multifocal and can mimic bacterial osteomyelitis or neoplastic processes. The clavicle, scapula, ribs, sternum, and vertebrae, are the most frequently affected bones, whereas calvarial involvement is extremely rare. A systematic search of several databases (Cochrane Library, LILACS, MEDLINE, PubMed, PubMed Central, and SciELO) revealed only a few case reports of cranial bone involvement, all in immunocompromised patients such as individuals with systemic lupus erythematosus. To our knowledge, such cases have not been reported in immunocompetent individuals^5^ ^-^ ^7^.

This report describes a rare case of PCM involving an osteolytic lesion of the calvarium in an immunocompetent adult with histopathological confirmation and a favorable clinical response to antifungal therapy. The unusual anatomical site in a patient with no known immunosuppression and the diagnostic challenges it posed, underscore the importance of considering PCM in the differential diagnosis of cranial osteolytic lesions, even in atypical clinical contexts. 

## CASE REPORT

**FIGURE 1:**
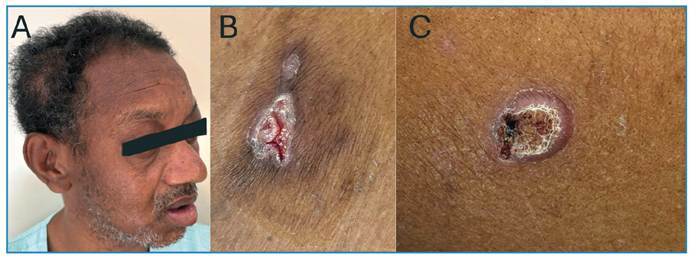
**(A)** Swelling in the right parietal region. **(B)** Cutaneous ulcer with well-defined borders and a granular base in the lateral region of the left 9th-10th costal arch. **(C)** Ulcerated plaque on the lateral middle third of the left arm, with raised and well-defined borders, a granular base with hemorrhagic dots, and no evident exudate.

**FIGURE 2:**
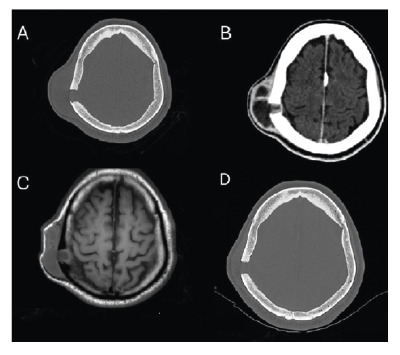
**(A)** Brain computed tomography, bone window, axial, showing a right parietal lytic bone lesion affecting the entire thickness of the shell: external board, diploe and internal board. There is also enlargement of adjacent soft tissues. **(B)** Brain computed tomography, parenchyma window, axial, showing fluid accumulation with peripheral enhancement in the soft tissues and involving the bone plane. **(C)** Brain magnetic resonance imaging (axial T1) showing fluid accumulation (hypointense on T1) in the soft tissues and in the bone of the right parietal region. **(D)** Brain computed tomography, bone window, axial, showing evident regression of adjacent soft tissues in the right parietal region after only three months of treatment.

A 47-year-old man from an urban area in southeastern Brazil was referred to a tertiary hospital with a progressively enlarging parietal mass, reaching approximately 6 cm in diameter over six months (Figure 1). During this period, the patient experienced weight loss exceeding 10% of his baseline body weight. Brain computed tomography revealed a right parietal lytic bone lesion involving the entire thickness of the calvarium, with adjacent soft tissue swelling, fluid accumulation, and peripheral contrast enhancement (Figure 2). 

The patient was admitted for further investigation with a preliminary suspicion of neoplasia. Additional laboratory investigations were conducted to rule out alternative etiologies, including serum lactate dehydrogenase, carcinoembryonic antigen, beta-human chorionic gonadotropin, alpha-fetoprotein, serum and urinary protein electrophoresis, and beta-2 microglobulin. Autoimmune screening for antinuclear antibody, quantitative immunoglobulin levels (IgA, IgG, and IgM), and serological testing for HIV, hepatitis B and C, and syphilis were also performed. All results were within the normal reference ranges.

**FIGURE 3:**
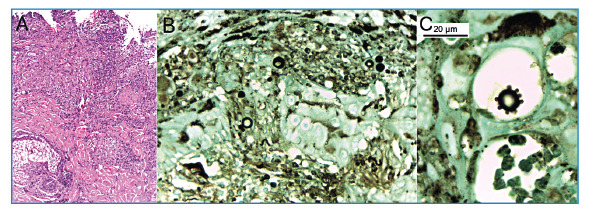
**(A)** Histology showing chronic granulomatous ulcerated dermatitis with microabscesses. Hematoxylin-eosin staining; magnification, ×100. **(B)** Yeast-like fungal structures of varying diameters. Grocott methenamine silver staining; magnification, ×400. **(C)** Oil-immersion image highlighting a fungal structure with multiple narrow-based budding daughter cells. Grocott methenamine silver staining; magnification: ×1000.

A thorough physical examination revealed two skin ulcers with raised, well-defined borders on the chest and left arm (Figure 1). A dermatological consultation was requested, and direct examination of a smear of the skin lesions with 10% potassium hydroxide revealed yeast-like fungal structures. Histopathological analysis revealed dermal granulomas and fungal structures with multiple budding forms on Grocott staining consistent with *Paracoccidioides* spp. (Figure 3). Bacterial, mycobacterial, and fungal cultures were negative, whereas serological results for PCM were positive.

Despite a 28-day hospitalization, a definitive diagnosis was established only after a dermatological evaluation. The patient was subsequently discharged with 200 mg/day of itraconazole. The patient exhibited significant clinical improvement, with complete healing of the ulcerated lesions on the chest and left arm, and progressive reduction of the parietal mass. Serological testing for total antibodies against *Paracoccidioides* spp. showed a titer of 1:16 prior to the initiation of antifungal therapy, which declined to 1:4 at two months and became nonreactive after six months of treatment.

Although a biopsy of the parietal bone lesion was not performed because of the invasiveness of the procedure, the marked radiological improvement observed after only three months of antifungal therapy (Figure 2) strongly supported the interpretation that the cranial lesion shared the same etiological agent as the cutaneous lesions.

## DISCUSSION

Osseous involvement in PCM is rare and primarily affects both children and young adults. PCM typically manifests as osteolytic lesions, usually without periosteal involvement or marginal sclerosis, and predominantly involves the long bones. Skull bone involvement has been rarely reported in the literature, with documented cases mainly involving transplant recipients. Bone involvement may lead to secondary osteomyelitis and soft-tissue infections^8^.

An early and accurate diagnosis is crucial for preventing long-term complications. A high index of clinical suspicion combined with fast and cost-effective diagnostic methods is essential for timely management. Accurate diagnosis relies on clinical and epidemiological correlations supported by complementary tests such as mucocutaneous lesion biopsy, fungal culture, and direct fungal examination, which typically reveal round budding yeast cells^9^.

Treatment was continued until the cure criteria were met, with itraconazole as the first-line therapy because of its superior efficacy and shorter treatment duration. Sulfamethoxazole-trimethoprim is an alternative, whereas amphotericin B is reserved for severe cases. Voriconazole is recommended for central nervous system involvement^10^ ^,^ ^11^. Follow-up should extend for at least two years post-cure and include clinical, radiological, serological, and mycological assessments. Monitoring is advised for at least one year after serological negativization^12^.

The characteristics of this case indicate that PCM should be considered in the differential diagnosis of skeletal lesions, particularly in patients from endemic regions or those with relevant epidemiological exposure^12^.

## Data Availability

Research data is available in the body of the document (References).
